# Leukemia Inhibitory Factor Is Required for Subventricular Zone Astrocyte Progenitor Proliferation and for Prokineticin-2 Production after a Closed Head Injury in Mice

**DOI:** 10.1089/neur.2020.0063

**Published:** 2021-06-25

**Authors:** Michelle J. Frondelli, Steven W. Levison

**Affiliations:** Department of Pharmacology, Physiology and Neuroscience, New Jersey Medical School, Rutgers University, Newark, New Jersey, USA.

**Keywords:** astrogliosis, cell proliferation, cytokines, neurotrauma, stem cells

## Abstract

Astrogliosis is one of the hallmarks of brain injury, and after a mild injury activated astrocytes subserve neuroprotective and pro-regenerative functions. We previously found that the astroglial response to closed head injury (CHI) was blunted in mice that were haplodeficient in leukemia inhibitory factor (LIF); therefore, the goal of these studies was to determine if the delayed astrogliosis was due to decreased recruitment of subventricular zone (SVZ) progenitors. CHI's were performed on post-natal day 20 on LIF heterozygous (Het) and wild-type (WT) mice. At 48 h post-CHI, astrocyte progenitor proliferation within the SVZ increased ∼250% in WT mice but was reduced by ∼200% in LIF Het mice compared with sham controls. Using neurospheres to model the SVZ, LIF increased the percentage of proliferating astrocyte progenitors by 2-fold compared with controls but had no effect on neural stem cell proliferation. To rule out the involvement of other cytokines, 105 cytokines were analyzed using a multi-plex array and with targeted validation on injured LIF Het versus WT neocortex. Of the cytokines analyzed, only prokineticin-2 (ProK2) required LIF signaling. Correspondingly, LIF-treated neurospheres expressed higher levels of ProK2, the ProK1 and ProK2 receptors (ProKR1 and ProKR2). Using *in situ* hybridization, ProK2 messenger RNA (mRNA) was most abundant in neocortical neurons and highly expressed within the SVZ. However, in contrast to LIF, ProK2 decreased astrocyte progenitor proliferation 2-fold. Altogether, these data demonstrate that LIF is necessary for astrocyte progenitor proliferation after injury and reveal a new role for LIF as an essential regulator of the neurotrophic factor ProK2.

## Introduction

Traumatic brain injury (TBI) affects 2.87 million people annually in the United States alone, and of these, 30% are in children under the age of 14.^1^ Pediatric TBI is especially detrimental because it can alter the trajectory of brain development. An important consideration is that it changes the proliferation of astrocyte progenitors, whose progeny participate in synaptogenesis, synaptic pruning, and the formation of peri-neuronal nets.^2–4^ Perturbances in gliogenesis can contribute to motor impairments, intellectual disabilities, and disturbed emotional behaviors that are post-TBI morbidities.^5^ Therefore, it is essential to establish how head injury affects gliogenesis.

A number of cytokines are produced subsequent to a TBI that participate in tissue remodeling. Among them is leukemia inhibitory factor (LIF), which surges as a result of brain injury.^6^ LIF reduces injury progression through its neurotrophic actions, promotes myelination, and activates astrocytes.^6–9^ Additional studies have found that LIF acts directly on the neural stem cells (NSCs) of the subventricular zone (SVZ) to promote NSC self-renewal as well as expansion of the NSC pool.^10,11^ In our studies of pediatric TBI using a controlled cortical impact (CCI) model, we documented a 2-fold increase in NSCs that correlated with a 15-fold increase in LIF levels.^6,12^ However, those studies did not establish whether LIF is required for the increase in NSCs.

Our previous studies also found that the astroglial response to closed head injury (CHI) is altered in LIF heterozygous (Het) mice. At 2 days post-injury (DPI), wild-type (WT) mice that had received a closed head injury at post-natal day 20 (P20) showed a 2-fold increase in glial fibrillary acidic protein (GFAP) staining in the neocortex and corpus callosum (CC) when compared with sham controls. By contrast, GFAP levels were unchanged in the LIF Het injured mice.^6^ This lack of response by the astrocytes correlated with increased white matter (WM) cell death and increased axonal injury at early time-points. Astrogliosis is complex, thus the failure of the astrocytic response could have arisen from the failure of the resident astrocytes to respond to the injury as well as to the failure of the progenitors within the SVZ to become activated and to generate new astrocytes. As we have found that the response of the SVZ cells is altered in LIF Het mice in response to a peri-natal hypoxic-ischemic injury,^13^ here we set out to test the hypothesis that LIF is required for NSC and astrocyte progenitor activation in the SVZ after a CHI.

## Methods

### WT and LIF heterozygous mice

The animal protocol was approved by the New Jersey Medical School Institutional Animal Care and Use Committee (IACUC) (#999900841) and was in accordance with the National Institutes of Health (NIH) Guide for the Care and Use of Laboratory Animals (Publication #80-23). CD-1 mice were injured on P20, the age roughly equivalent to humans age 2–3 years old.^14^ LIF levels peak at 48 h post-injury (PI), therefore, both WT and LIF Het mice were administered a CHI and terminated 48 h later.^6^ LIF Het mice were produced from a CD-1 mouse line, in which the gene encoding LIF was mutated by disrupting the third exon that truncated the LIF protein so that it was missing the last carboxyterminal 81 amino acids, including the last nine, that are essential for its biological activity.^15^ The germline LIF Het mice used for these studies were maintained on a CD1 background. LIF Het male mice were crossed with either LIF Het or WT females; thus, littermates were used for these studies. Mice were genotyped as described previously.^16^

### Closed head injury

The eCCI Model 6.3 (Cat. #23298-047; Custom Design & Fabrication) driven-piston impactor produced a bilateral CHI, midway between bregma and lambda. Mice were anesthetized, a midline incision was created, and an impactor tip of 5 mm in diameter created the CHI on the intact skull at a velocity of 4 m/sec to a depth of 1 mm with a dwell time of 150 msec. Age-matched sham-operated animals received the same isoflurane treatment and scalp incisions. Mice were monitored for 2 h after surgery and received buprenorphine, subcutaneously, as post-operative care at 0.05 mg/kg. Mice were returned to their cages and checked daily until terminated by intracardiac perfusion with 3% paraformaldehyde (PFA) in phosphate-buffered saline (PBS).

### Coronal sections and immunostaining

The brains were frozen in Tissue-Tek optimal cutting temperature (OCT) matrix embedding medium and sectioned coronally at 40 μm, using a Leica CM1950 cryostat. Sections were immunostained using rabbit anti-glutathione S-transferase-1 (GST-mu1) YB1 (1:100, Cat. #Bio28YB1, Biotrin) and rat anti-Ki-67(1:250, Cat. #1456882, eBioscience). The following secondary antibodies were used: Alexa 488-conjugated donkey anti-rabbit (Cat. #711-485-152, Jackson ImmunoResearch) and Cy3-conjugated donkey anti-rat (Cat. #715-506-153, Jackson ImmunoResearch) used at 1:200 dilution using a protocol described previously.^6^

### Stereology

To quantify GSTmu1+/Ki-67+ cells, sections were analyzed using the MicroBrightField Stereo Investigator program and an Olympus BX51 microscope. Slides were coded so that the researcher was blinded to the genotype and injury condition. Each region was traced, and randomly placed counting frames were applied within the traced region at predetermined regular X and Y intervals. Twenty percent of the SVZ was counted, as determined necessary using Cavalieri point counting equations, including profile area and profile boundary equations. Image stacks were captured at 100 × using an immersion oil objective. Selection criteria for counting an object within the sampling frame followed stereological counting rules.^17^

### Flow cytometry on neurospheres

Neurospheres were produced from P4, WT CD1 mice and propagated in growth medium consisting of Dulbecco's modified Eagle's medium (DMEM)/F-12 supplemented to 15 mM 4-(2-hydroxyethyl)-1-piperazineethanesulfonic acid (HEPES), 1% GlutaGro (Cat. #25-015-Cl, Corning), 1% Antibiotic-Antimycotic (Cat. #1540062, ThermoFisher), 2% B27 supplement (Cat. #17504001, ThermoFisher), 20 ng/mL recombinant human epidermal growth factor (EGF) (Cat. #AF-100-15, PeproTech), 10 ng/mL recombinant human fibroblast growth factor (FGF)-2 (Cat. #100-18C, PeproTech), and 1 ng/mL heparin sulfate. Cells were maintained at 37°C with humidified 5% CO_2_ to permit primary neurosphere formation and were fed every 2 days until they reached ∼200 μm in diameter.^18^ The cells were deprived of growth factors for 15 h. Growth factors were reintroduced to the cultures ± recombinant murine LIF (Cat. #250-02, PeproTech) at 5 ng/mL or recombinant murine ProK2 (Cat. #315-38, PeproTech) at 1 × 10^−8^ M. After 24 h, 5-ethynyl-2′-deoxyuridine (EdU) was added to cell cultures for 24 h at 10 μM, except one plate that was deprived of EdU, serving as a negative control.

Cells were collected and incubated with 2.6 Wünsch units/mL of LiberaseDH and 20 μg/mL of DNAse in DMEM/F-12 and incubated at 37°C for 5 min with intermittent stirring. Enzymatic digestions were quenched with an equal volume of DMEM/F-12 containing 20 μg/mL DNAse. The cells were centrifuged for 5 min at 300 × *g* and dissociated by repeated trituration. Cells were centrifuged, counted, diluted to 1 million cells/mL in DMEM/F-12, and stained using the antibodies listed in Table 1.

**Table 1.** List of Primary and Secondary Antibodies Used to Complete Flow Cytometry Studies

Antibody/Reagent	Dilution	Host	Isotype	Fluorophore	Clone	Catalog #	Vender
LIVE/DEAD	1:1000	NA	NA	Blue	NA	L34961	Thermo Fisher
CD15 (LeX)	1:20	Mouse	IgM	V450	MC480	561561	BD Biosciences
CD140 (PDGFRα)	1:160	Rat	IgG2a, K	PE	APA5	135906	Biolegend
NG2	1:50	Rabbit	IgG	NA	NA	AB5320	Millipore
Goat anti-rabbit	1:100	Goat	NA	A700	NA	A21038	Life Technologies
GLAST (biotin)	1:11	Mouse	IgG2a, K	NA	ASCA-1	130-118-984	MACs (Miltenyi)
Strepavidin APC	0.125 ug/test	NA	IgG2a, K	780	NA	47-4317-82	eBioscience
O4	1:125	Mouse	IgG1	APC	REA576	130-119-897	MACs (Miltenyi)
CD133	0.01 ug/test	Rat	IgG1, K	Superbright 600	13A4	63-1331-80	Thermo Fisher
FcR Block	1:20	NA	NA	NA	NA	30-092-575	MACs (Miltenyi)
Click-It Plus EdUAlexa Fluor Flow Cytometry Assay Kit	NA	NA	NA	488	NA	C10633	Thermo Fisher

### Quantitative polymerase chain reaction

Damaged neocortices of CHI mice were dissected at P22. RNA was extracted by homogenenization in Trizole^TM^ (Cat. #15596018, Thermo Fisher), followed by chloroform, isopropanol extractions, and ethanol precipitation. RNA was quantified using an ND-1000 spectrophotometer (Nanodrop, Thermo Fisher) and samples were diluted to 50 ng/μL. Complementary DNA (cDNA) was synthesized using Superscript II reverse transcriptase (Cat. #18064014, Thermo Fisher) using a MultiGene Optimax machine (Labnet International). All primers, housekeeping genes, and corresponding master mixes were purchased from Qiagen. Blanks were included to establish the background signal and 75 ng of universal mouse reference RNA (Cat. #14920, ThermoFisher) served as a positive control. Samples were run for 40 cycles as follows: 50°C for 10 min, 95°C for 5 min, 95°C for 10 sec, and 60°C for 30 sec. The plates were read using a CFX96 Real-Time quantitative polymerase chain reaction (qPCR) detection system (BioRad) and the data were analyzed using CFX Manager Software (BioRad).

### qPCR Bioplex Assay

Damaged neocortices of CHI mice were dissected at P22. Actin was chosen to standardize the quantitative experiments. The differences between samples were calculated as the specific ratio of the gene of interest/actin. Samples were prepared using RT^2^ SYBR Green qPCR Mastermix (Cat. #330504 Qiagen) and analyzed using a RT^2^ Profiler PCR Mouse Growth Factor Array (Cat. #330231 Qiagen), according to manufacturer's instructions. All samples were run using a total of 750 ng of cDNA per plate. Samples were run for 40 cycles as follows: 95°C for 15 sec, then 60°C for 60 sec. Plates were read using a CFX96 Real-Time PCR Detection System and analyzed using CFX Manager Software.

### ELISA

Damaged neocortices of CHI mice were dissected at P22. Samples were collected and processed in radioimmunoprecipitation (RIPA) buffer. Tissue was homogenized, rested on ice for 10 min, and sonicated for 5-sec pulses 3 times, and returned to ice for 10 min. Samples were centrifuged at 300 × *g* for 15 min at 4°C and protein concentration was measured using a Pierce BCA Kit (Cat. #23227, ThermoFisher). Absorbance was measured at 570 nm. ProK2 protein was quantified by indirect enzyme-linked immunosorbent assay (ELISA) (Cat. #ABIN5521865, Antibodies-online) using 100 mg of sample protein.

### Paraffin sections and RNAScope

Mice were perfused with 3% PFA in UltraPure DNAse/RNAse-free distilled water (Cat. #10977015, Invitrogen). Brains were post-fixed overnight in 3% PFA, dehydrated through alcohols, and embedded in paraffin. Coronal sections at 6 μm were mounted onto Superfrost Plus slides. Sections were deparaffinized in xylene, washed in 100% ethanol, and dried at 65°C for 5 min. Then the sections were incubated with 100% hydrogen peroxide for 10 min, and washed 3 times. Sections next were steamed at 99°C for 15 min, encircled with a barrier pen, hybridized with a probe, and then incubated with amplifiers (Cat. #323136 and #447941, Advanced Cell Diagnostics). The fluorescence signal was developed using a fluorescent horseradish peroxidase substrate (Cat. #FP1488001KT, Akoya Biosciences). Following *in situ* hybridization, sections were counterstained with NeuroTrace 500/525 Green Fluorescent Nissl Stain (Cat. #N21480, ThermoFisher) and then sealed using Prolong Gold Antifade Mountant with DAPI. Images were acquired using a Keyence BZ-9000 All-in-one Fluorescence Microscope.

### Statistical analysis

Statistical analyses were performed using GraphPad Prism Software. The means between two groups were compared using two-tailed, unpaired *t* test. The means between more than two groups were compared using one-way or two-way analysis of variance (ANOVA) with Tukey's post hoc test. Error bars denote standard deviations (SDs). Comparisons were interpreted as significant when associated with *p* < 0.05.

## Results

### Several growth factors are increased in WT mice when compared with LIF Het CHI mice at 48 h after CHI

In general, increases in cell proliferation are due either to increases in specific growth factors or decreases in growth inhibitors. Therefore, to determine whether the signals that regulate cell proliferation were different between WT injured and LIF Het injured mice, we used the commercially available RT^2^ Profiler PCR Mouse Growth Factor Array, which enabled us to simultaneously screen 84 growth factors. As these are diffusible signals, we hypothesized that they would be produced by cells in the damaged neocortex and underlying WM, whereupon they would spread to the SVZ. As our goal was to identify transcripts that were differentially expressed after injury in the LIF Hets versus the WT, we chose to perform a qPCR Bioplex Assay only on the WT CHI and LIF Het CHI groups at 2 DPI. Contrary to our expectations, only a few growth factors were significantly different between the LIF Het and the WT injured mice, including colony stimulating factor 3, FGF-4, FGF-14, c-fos induced growth factor, LIF, and transforming growth factor-β2 (Table 2).

**Table 2.** Growth Factors and Average Values at 48 h PI in the Neocortex

Cytokine/Growth Factor	WT injured relative value average	LIF Het injured relative value average	Ratio of WT injured: LIF Het injured	*P*-value
Anti-mullerian hormone	0.92	0.66	1.4	0.25
Artemin	40.64	24.78	1.64	0.27
Brain derived neurotrophic factor	43.13	27.89	1.55	0.15
Bone morphogenetic protein 1	221.64	128.64	1.72	0.03^*^
Bone morphogenetic protein 10	19.21	2.52	7.63	0.33
Bone morphogenetic protein 2	6.74	6.82	0.99	0.98
Bone morphogenetic protein 3	20.86	17.69	1.18	039
Bone morphogenetic protein 4	31.24	26.74	1.17	0.57
Bone morphogenetic protein 5	75.78	38.97	1.95	0.20
Bone morphogenetic protein 6	49.05	31.40	1.56	0.22
Bone morphogenetic protein 7	28.558	27.0917	1.054	0.813
Bone morphogenetic protein 8a	2.606	1.872	1.393	0.476
Bone morphogenetic protein 8b	18.84	17.48	1.08	0.90
Colony stimulating factor 1 (macrophage)	19.33	12.73	1.52	0.10
Colony stimulating factor 2 (granulocyte-macrophage)	23,288.99	14,453.81	1.61	0.31
Colony stimulating factor 3 (granulocyte)	7.15	2.96	2.41	0.04^*^
Chemokine (C-X-C motif) ligand 1	0.29	0.25	1.17	0.85
Chemokine (C-X-C motif) ligand 12	119.99	91.46	1.31	0.32
Epidermal growth factor	138.40	26.35	5.25	0.10
Epiregulin	36.43	28.53	1.28	0.60
Fibroblast growth factor 1	57.24	56.08	1.02	0.85
Fibroblast growth factor 10	69.60	43.17	1.61	0.15
Fibroblast growth factor 11	31.64	28.76	1.10	0.82
Fibroblast growth factor 13	613.25	496.74	1.24	0.34
Fibroblast growth factor 14	148.70	100.47	1.48	0.01^*^
Fibroblast growth factor 15	585.24	410.71	1.43	0.39
Fibroblast growth factor 17	4.33	3.08	1.40	0.31
Fibroblast growth factor 18	20.69	12.04	1.72	0.27
Fibroblast growth factor 2	9.84	7.64	1.29	0.52
Fibroblast growth factor 22	6.90	4.56	1.51	0.34
Fibroblast growth factor 3	60.78	41.15	1.48	0.30
Fibroblast growth factor 4	1.23	0.39	3.18	0.03^*^
Fibroblast growth factor 5	19.57	11.90	1.64	0.16
Fibroblast growth factor 6	0.50	0.38	1.32	0.16
Fibroblast growth factor 7	1.85	0.54	3.40	0.03^*^
Fibroblast growth factor 8	12.13	8.81	1.38	0.63
Fibroblast growth factor 9	41.78	36.30	1.15	0.62
C-fos induced growth factor	12.85	5.68	2.26	0.05^*^
Growth differentiation factor 10	28.90	26.50	1.09	0.75
Growth differentiation factor 11	79.50	85.70	0.93	0.72
Growth differentiation factor 5	3.06	2.04	1.50	0.36
Glial cell line derived neurotrophic factor	9.89	7.27	1.36	0.31
Hepatocyte growth factor	22.73	17.99	1.26	0.42
Insulin-like growth factor 1	44.91	25.51	1.76	0.19
Insulin-like growth factor 2	61.39	46.48	1.32	0.21
Interleukin 11	8.03	5.84	1.37	0.50
Interleukin 12A	13.98	10.03	1.39	0.39
Interleukin 18	56.41	50.80	1.11	0.58
Interleukin 1 alpha	30.86	19.04	1.62	0.11
Interleukin 1 beta	16.12	6.96	2.32	0.11
Interleukin 2	30.52	17.14	1.78	0.31
Interleukin 3	15.27	2.49	6.13	0.31
Interleukin 4	58.70	35.84	1.64	0.37
Interleukin 6	4.37	1.69	2.58	0.12
Interleukin 7	8.35	4.23	1.98	0.20
Inhibin alpha	93.78	82.38	1.14	0.65
Inhibin beta-A	122.90	86.24	1.43	0.24
Inhibin beta-B	21.74	47.96	0.45	0.44
Kit ligand	114.89	90.39	1.27	0.40
Left right determination factor 1	1.61	1.68	0.96	0.96
Left right determination factor 2	236.04	99.62	2.37	0.13
Leptin	10.86	7.88	1.38	0.45
Leukemia inhibitory factor	2.47	4.48	0.55	0.04^*^
Midkine	38.22	28.53	1.34	0.35
Myostatin	16.82	12.47	1.35	0.46
Nerve growth factor	89.49	59.01	1.52	0.38
Nodal	12.98	5.55	2.34	0.13
Neurotrophin 3	3.77	1.80	2.10	0.29
Neurotrophin 5	47.73	30.12	1.59	0.46
Platelet derived growth factor, alpha	29.59	27.24	1.09	0.74
Placental growth factor	18.26	9.70	1.88	0.34
Rabaptin, RAB GTPase binding effector protein 1	231.79	163.97	1.41	0.20
S100 calcium binding protein A6 (calcyclin)	122.25	73.75	1.66	0.14
Secreted phosphoprotein 1	262.91	34.25	7.68	0.38
Teratocarcinoma-derived growth factor 1	6.31	2.72	2.32	0.19
Trefoil factor 1	20.95	17.13	1.22	0.64
Transforming growth factor, alpha	14.14	11.08	1.28	0.19
Transforming growth factor, beta 1	44.53	33.47	1.33	0.11
Transforming growth factor, beta 2	11.24	6.11	1.84	0.02^*^
Transforming growth factor, beta 3	45.46	24.52	1.85	0.17
Vascular endothelial growth factor A	159.40	117.04	1.36	0.24
Vascular endothelial growth factor B	101.43	115.29	0.88	0.52
Vascular endothelial growth factor C	6.49	3.94	1.65	0.40
Zinc finger protein 91	220.833	180.771	1.222	0.15

This table lists the growth factors/cytokines analyzed using the RT^2^ Profiler PCR Mouse Growth Factor Array. Values were normalized to actin for WT injured and LIF Het injured mice at 2 days of recovery from CHI. The ratios of WT injured to LIF Het Injured are provided at *n* = 3 mice per group. Statistical comparisons were performed using a two-tailed, unpaired Student's *t* test and Tukey's post hoc test. ^*^p < 0.05 by Student's *t* test and Tukey's post hoc test.

CHI, closed head injury; Het, heterozygous; LIF, leukemia inhibitory factor; WT, wild-type.

### Several cytokines are dysregulated with LIF deficiency after CHI

Using the criterion of a 2.5-fold difference, several cytokines of interest from the array were selected for validation using qPCR, including bone morphogenetic protein-10, EGF, FGF-4, FGF-7, interleukin (IL)-3, IL-6, and secreted phosphoprotein-1 (Table 3). Some additional molecules were added for analysis including cyclooxygenase-2 and GFAP.^19^ Galanin was selected, as previous studies had shown that galanin is regulated by LIF and that it promotes oligodendrocyte cell survival.^20^ Neurotrophin-3 was examined for its known ability to promote oligodendrocyte survival.^21^ Studies had shown that ProK2 directionally attracts SVZ cells, suggesting a regenerative function in the injured cortex; therefore, we analyzed ProK2.^22^ We also analyzed the ProK2 receptors (ProKR1 and ProKR2). Vascular endothelial growth factor-A was examined for its known effects on astrocytes, as revealed in the LIF null mouse.^23^ Endothelin-1 was analyzed for its known loss after an increase in neurogenesis and its known effect of being produced by astrocytes in culture and in the injured brain.^24,25^ Erythropoietin was analyzed because it is known to increase proliferation and maturation of oligodendrocytes.^26^ Of these (excluding ProK2) only the cytokine IL-1α was significantly different between WT injured and the WT sham mice.

**Table 3.** Average Relative Values of Cytokines in the Cerebral Cortex at 48 h after CHI

Cytokine	LIF Het injured average	WT injured average	LIF Het sham average	WT sham average
BMP6	3.06	4.21	3.31	4.10
BMP10	3.97	10.16	5.91	3.43
COX2	3.53	3.25	3.45	3.59
END1	2.59	2.47	2.77	3.47
EGF	10.56	19.71	10.88	12.39
EPO	12.90	17.81	18.58	14.48
FGF4	5.14	4.84	4.57	2.74
FGF7	8.27	19.26	15.95	12.70
GALANIN	11.33	7.28	9.91	7.28
GFAP	10.77	6.98	1.86++	1.43
IGF1L	1.94	1.86	1.89	1.58
IGF1R	5.83	4.24	4.54	5.71
IL1A	5.52	6.49	2.95	3.32#
IL6	3.36	6.07	6.80	5.07
NT3	3.05	2.99	1.85	2.40
SPP1	3.70	25.21	0.93	1.38
VEGFA	1.03	9.49	7.32	0.90

WT and LIF Het mice were injured at P20. After 2 days, the injured cortex from each animal was microdissected, RNA was isolated, and cDNA was synthesized. Gene expression was analyzed by qPCR. Values represent expression levels as normalized to actin; *n* = 3–6 animals per group. Statistical significance was evaluated by two-way ANOVA and Tukey's post hoc test. + denotes difference between LIF Het injured and LIF Het sham mice. # denotes differences between WT injured and WT sham mice.

ANOVA, analysis of variance; cDNA, complementary DNA; CHI, closed head injury; Het, heterozygous; LIF, leukemia inhibitory factor; P, post-natal day; qPCR, quantitative polymerase chain reaction; WT, wild-type.

### SVZ astrocyte progenitor cell proliferation is depressed in the LIF Het mice at 48 h post-CHI

**FIG. 1.**
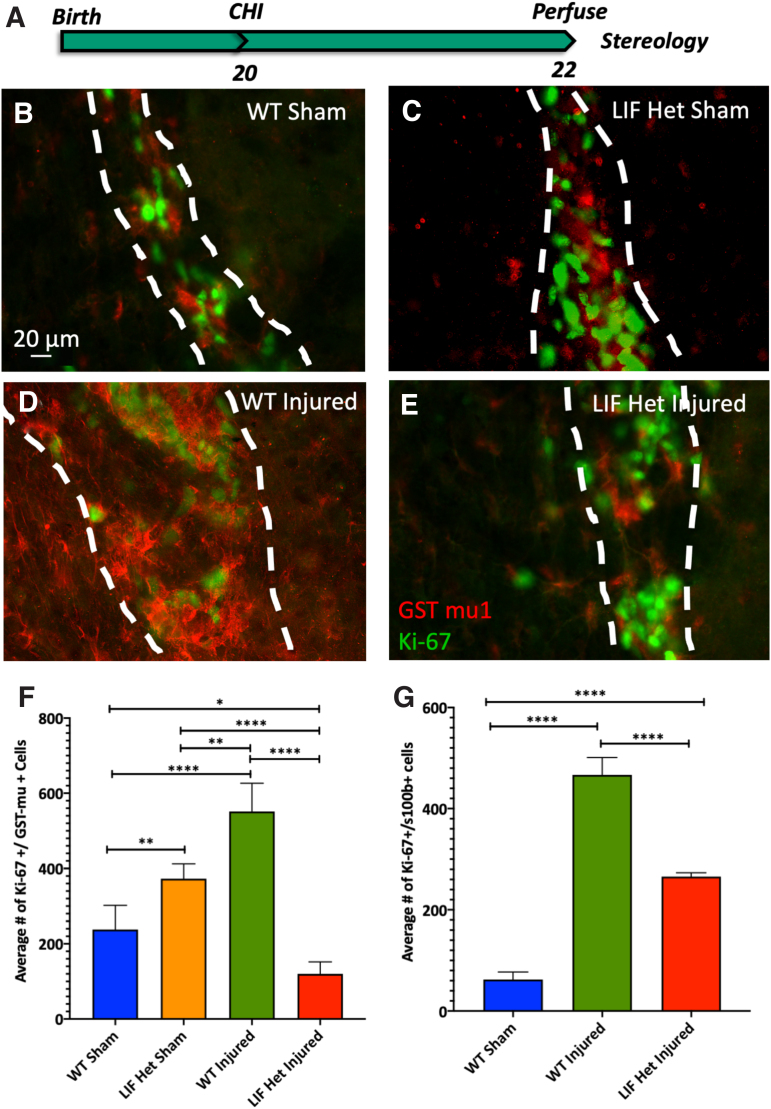
SVZ astrocyte progenitor cell proliferation is depressed in the LIF Het mice at 48 h post-CHI. **(A)** Schematic of experimental design. WT and LIF Het mice were injured at P20 and analyzed at 2 DPI. Coronal 40-μm sections containing the SVZ were stained for Ki-67 and GST-mu1. **(B–E)** Representative images from the SVZ below the center of the impact zone 2 DPI from (B) WT sham, (C) LIF Het sham, (D) WT injured, and (E) LIF Het injured mice. Dotted white lines outline the SVZ. **(F)** Stereological counts of Ki-67+/GST-mu+ cells in the SVZ from WT sham, LIF Het sham, WT injured, and LIF Het injured mice. *p ≤ 0.05; **p < 0.01; ***p < 0.001; ****p ≤ 0.0001 by two-way ANOVA and Tukey's post hoc test. Values represent means ± SD from *n* = 5 animals per group. **(G)** Stereological counts of Ki-67+/S100b+ cells in the WM from WT sham, WT injured, and LIF Het injured mice. ****p < 0.0001 by one-way ANOVA and Tukey's post hoc test. Values represent means ± SDs from *n* = 3 animals per group. ANOVA, analysis of variance; CHI, closed head injury; DPI, DPI = days post-injury; Het, heterozygous; LIF, leukemia inhibitory factor; P, post-natal day; SD, standard deviation; SVZ, subventricular zone; WM, white matter; WT, wild-type.

GST-mu1 is a cytosolic enzyme that is expressed by a subset of cells within the SVZ and is expressed exclusively by WM and gray matter astrocytes, especially during early central nervous system (CNS) development.^27,28^ Further, previous studies by Cammer and Zhang^27^ found that GST-mu1 stains astrocyte progenitors near the ventricles and that as they mature, they gain GFAP staining. Therefore, we analyzed the GST-mu1+ cells in WT and LIF Het mice that were injured at P20 (Fig. 1A). Representative images depicting Ki-67+/GST-mu1+ cells in the SVZ at 2 DPI for each group are provided in Figure 1B–E. Using the optical fractionator, we quantified the numbers of GST-mu1+/Ki-67+ cells in the SVZ. There were twice as many double-positive cells in the WT injured SVZ when compared with WT sham, which was 4.5-times higher than the number of double-positive cells in the LIF Het injured mice (*p* < 0.0001) at 48 h PI (Fig. 1F). Surprisingly, the LIF Het sham mice had 3-fold more Ki-67+/GST-mu1+ cells compared with LIF Het injured mice at 48 h PI (*p* < 0.0001). Decreased astrocyte lineage cell proliferation also was observed in the white matter immediately overlying the SVZ where the WT injured mice had 7.5-fold (*p* < 0.0001) more Ki67^+^/S-100b^+^ cells compared with WT sham mice and the LIF Het injured mice had 50% fewer Ki67+/S-100b+ cells than the WT injured mice (*p* < 0.0001) (Fig. 1G).

### LIF promotes the proliferation of astrocyte progenitors

**FIG. 2.**
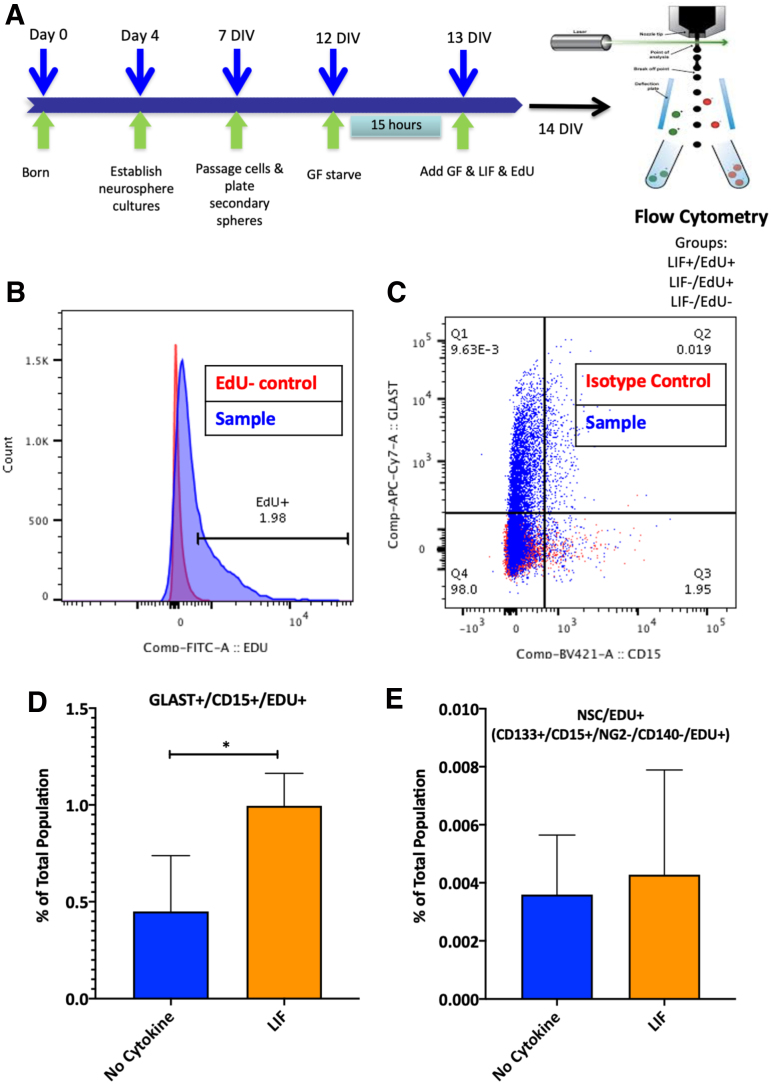
LIF promotes the proliferation of astrocyte progenitors. **(A)** Secondary neurospheres that had been propagated in growth medium containing 20 ng/mL EGF and 10 ng/mL FGF-2 were treated with 5 ng/mL LIF and incubated EdU for 24 h prior to flow cytometry analysis. **(B)** Sample of EdU+ gating based on EdU– control. **(C)** Sample of gating strategy of Glast+/CD15+ cells based on isotype control. **(D)** The percentage of Glast+/CD15+/EdU+ cells quantified between the two treatment groups. **(E)** The percentage of NSCs, defined as CD133+/LeX+/NG2–/CD140a– was quantified between the two treatment groups. Data are expressed as means ± SDs from three independent cultures per group. Statistical analyses were performed using Student's *t* test. *p ≤ 0.05. EdU, 5-ethynyl-2′-deoxyuridine; EGF, epidermal growth factor; FGF, fibroblast growth factor; LIF, leukemia inhibitory factor; NSC, neural stem cell; SD, standard deviation.

The data in Figure 1 suggest that LIF enhances the proliferation of astrocyte progenitors in the SVZ, and therefore, when LIF levels are depressed, there is less astrocyte progenitor cell proliferation in vitro. To test this hypothesis, we evaluated the effect of LIF on astrocyte progenitor cell proliferation. For this experiment, secondary neurospheres were deprived of growth factors for 15 h, whereupon EGF and FGF-2 were reintroduced to the cultures along with EdU ±5 ng/mL LIF. We then quantified the proportion of EdU+ astrocyte progenitors by flow cytometry (Fig. 2A). Cells were gated on EdU+ cells (Fig. 2B) and then gated on Glast+/CD15+ cells (Fig. 2C).^29^ LIF addition to neurosphere cultures increased the percentage of Glast+/CD15+/EdU+ cells 2-fold over controls, indicative of proliferating astrocyte progenitors (Fig. 2D). Although Glast primarily labels astrocyte lineage cells, it also is expressed by the NSCs in the SVZ; therefore, we analyzed EdU+ NSCs (CD133+/CD15+/NG2–/CD140–) and found that there was no significant difference in EdU incorporation between the two groups (Fig. 2E).^30^ These data are consistent with the interpretation that LIF promotes the proliferation of astrocyte progenitors and not the NSCs.

### Neocortical ProK2 protein levels increase in the neocortex by 96 h PI

**FIG. 3.**
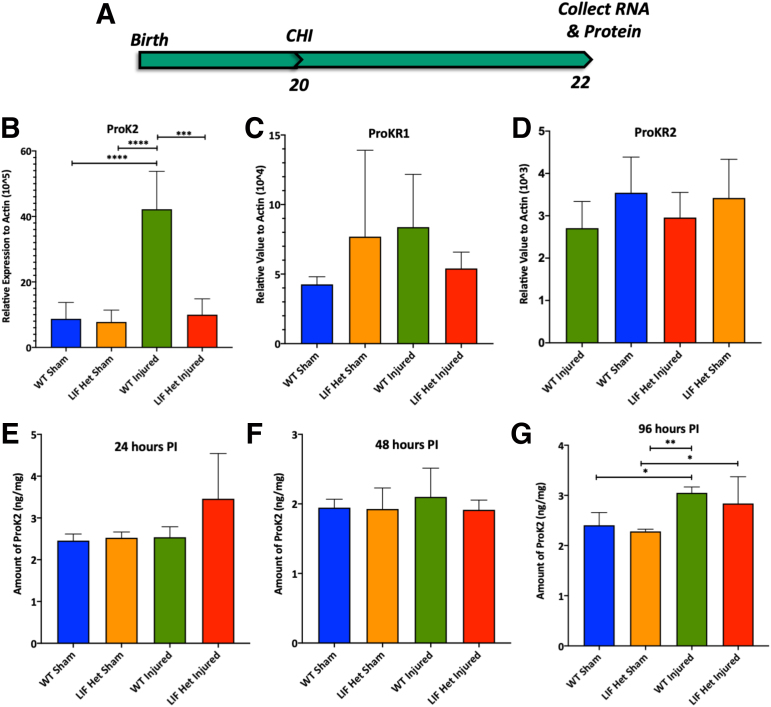
Neocortical ProK2 expression increases after CHI. **(A)** Schematic of experimental design. WT and LIF Het mice were injured at P20. After 2 days, injured cortex from each animal was microdissected, RNA was isolated, and cDNA was synthesized or protein was collected and processed for ELISA. **(B)** ProK2 was measured by qPCR and normalized to actin. **(C,D)** ProKR1 and ProKR2 levels were measured by qPCR. After 1, 2, or 4 DPI, the injured cortex from each animal was microdissected, protein was extracted, and an ELISA was performed at **(E)** 24 h PI, **(F)** 48 h PI, and **(G)** 96 h PI. Data represent means ± SDs from *n* = 5 animals per group. All tests performed by two-way ANOVA and Tukey's post hoc test. *p < 0.05; **p < 0.01, ****p < 0.0001. ANOVA, analysis of variance; cDNA, complementary DNA; CHI, closed head injury; DPI, DPI, days post-injury; ELISA, enzyme-linked immunosorbent assay; Het, heterozygous; LIF, leukemia inhibitory factor; P, post-natal day; PI, post-injury; ProK2, prokineticin-2; ProKR1, prokineticin-1 receptor; ProKR2, prokineticin-2 receptor; qPCR, quantitative polymerase chain reaction; SD, standard deviation; WT, wild-type.

Studies show that cells expressing ProK2 *in vitro* directionally attract SVZ cells and that it can stimulate the proliferation of astrocytes; therefore, ProK2 may participate in directing astrocyte progenitors toward areas that have sustained brain damage.^22,31^ Thus, we analyzed differences in levels of expression of ProK2 in WT and LIF Het mice in the injured and uninjured neocortex at 48 h PI (Fig. 3A). Messenger RNA (mRNA) levels were 5-fold higher in WT injured mice when compared with LIF Het injured mice and sham controls (*p* < 0.0001) (Fig. 3B) and there was no increase in ProK2 in the LIF Het injured versus LIF Het sham mice.

As ProK2 data showed differences between groups, we also evaluated the levels of ProKR1 and ProKR2 to establish which receptor is present in the juvenile brain and to establish whether levels of the receptors change with injury. qPCR analysis for ProKR1 (Fig. 3C) and ProKR2 (Fig. 3D) found that both receptors were expressed in the neocortex, with ProKR2 expressed ∼5-fold more than ProKR1. The levels of these receptors were not significantly altered by the injury. Next, we evaluated the protein levels of ProK2 by ELISA. We examined differences in protein levels at 24 h (Fig. 3E), 48 h (Fig. 3F), and 96 h (Fig. 3G) after CHI. This analysis failed to confirm an increase in ProK2 at 24 h and 48 h after CHI. However, protein levels lag behind changes in mRNA levels. At 96 h after CHI, WT injured mice showed a 30% increase in ProK2 compared with WT sham mice (*p* < 0.05), which also was 25% higher than levels of ProK2 in the LIF Het sham mice (*p* < 0.05) and LIF Het sham mice (*p* < 0.01).

### ProK2 inhibits the proliferation of Glast+ cells

Astrocytes express ProKR1 and ProK2 increases intracellular calcium levels in mouse astrocytes followed by an increase in proliferation.^31^ Because the number of proliferating astrocyte progenitors increased in WT injured mice and levels of ProK2 also increased in WT injured mice, we hypothesized that the lack of ProK2 in the LIF Het mice would lead to reduced astrocyte progenitor cell proliferation in the SVZ. To test this hypothesis, we performed an *in vitro* experiment using neurospheres to determine whether ProK2 would stimulate proliferation.

**FIG. 4.**
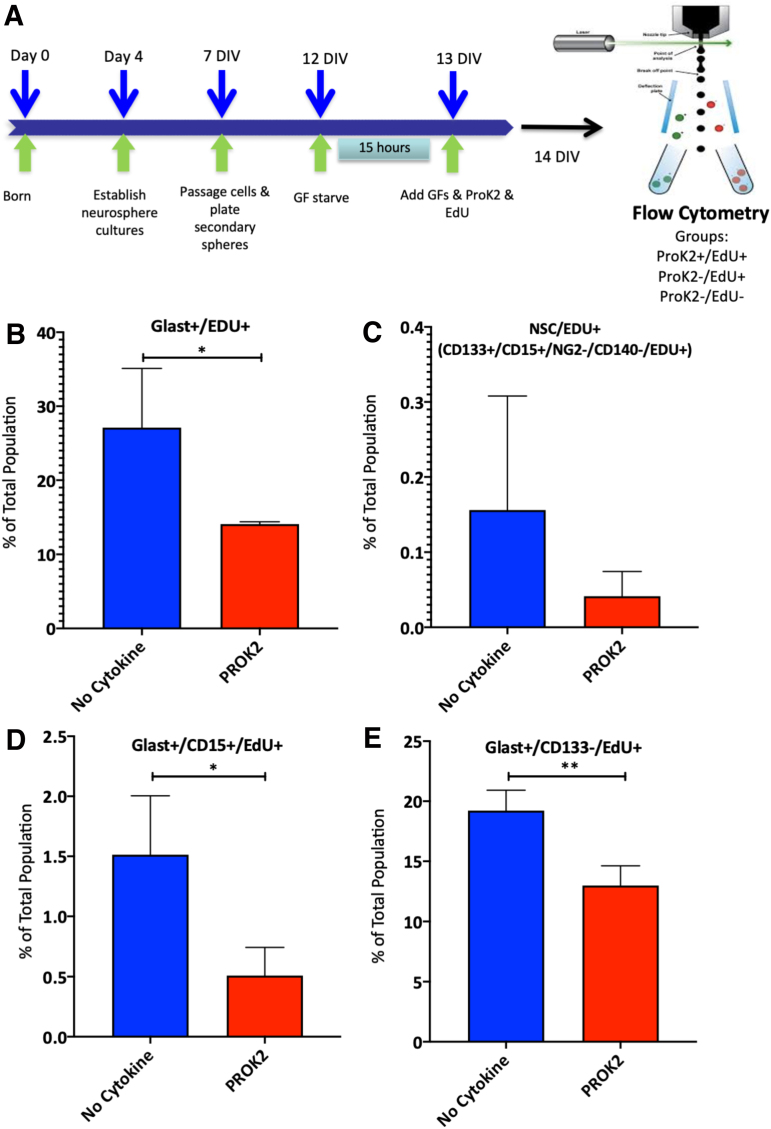
ProK2 inhibits astrocyte proliferation *in vitro*. **(A)** Schematic of experimental design. Secondary neurospheres were generated from P4, WT mice. The cells were passaged and starved of growth factors for 15 h to enhance cell cycle synchronization. Twenty-four hours later, 2 ng/mL EGF and 1 ng/mL FGF-2 were reintroduced ±1 × 10^–8^M recombinant murine ProK2 and EdU. Twenty-four hours later, the cells were stained for EdU and assessed by multi-color flow cytometry. The entire population of Glast+ progenitors was analyzed **(B)** as well as subpopulations including the NSCs **(C)** and two subsets of astroglial progenitors defined as Glast+/CD15+/CD133–/EdU+ **(D)** and Glast+/CD15–/CD133–/EdU+ **(E)**. Data are depicted as means ± SDs from three independent sets of cultures per group. Statistical analyses were performed using Student's *t* test. *p < 0.05; **p < 0.01. EdU, 5-ethynyl-2′-deoxyuridine; EGF, epidermal growth factor; NSC, neural stem cell; P, post-natal day; ProK2, prokineticin-2; SD, standard deviation; WT, wild-type.

The experimental design was identical to the experiment testing LIF, but here we used 1 × 10^−8^ M recombinant ProK2 treatment for 24 h (Fig. 4A). Contrary to our hypothesis, we found that ProK2 inhibited a variety of progenitors within the SVZ, which included the multi-potential progenitor-2 (MP2), platelet-derived growth factor and FGF-2 responsive multi-potential progenitor (PFMP), glial restricted progenitor 1 (GRP1), and GRP3 (Table 4). When the glial progenitors were analyzed further, there were 50% fewer Glast+/EdU+ cells in the cultures with ProK2 treatment (*p* < 0.05) (Table 5; Fig. 4B). Because Glast labels both astrocyte lineage cells as well as NSCs, we also analyzed the proliferating NSC population and found no significant differences in the proportions of EdU+ NSCs between controls and ProK2 treated groups (Fig. 4C). We subsequently analyzed two subpopulations of proliferating astrocyte progenitors: Glast+/CD15+/EdU+ cells and Glast+/CD133–/EdU+ cells. We found EdU incorporation was decreased by at least 30% in both subpopulations with the addition of ProK2 to the neurosphere culture medium (*p* < 0.05) (Fig. 4D,E). These data demonstrate that ProK2 inhibits the proliferation of astrocyte progenitors. ProK2 also inhibited the proliferation of the O4+ oligodendrocyte progenitors (*p* < 0.05).

**Table 4.** Average Percentages of Proliferating NSCs and Progenitors after 24 h of ProK2 Treatment or No Cytokine Treatment

	EdU+							
Group	NSC	MP1	MP2	BNAP/GRP1	MP4	MP3/GRP2	PFMP	GRP3
No cytokine average	0.160	0.083	0.426	0.619	0.566	38.242	0.092	7.889
ProK2 average	0.041	0.064	0.131^*^	0.339^*^	0.367	34.812	0.049^*^	3.991^*^

Neural tissue rich in SVZ cells were collected from P4 pups and neurosphere cultures were established. Spheres were propagated for several days, passaged, and grown for an additional 5 days. Spheres were starved of growth factors for 15 h and growth factors were reintroduced along with EdU and 1 × 10^−8^ M ProK2 or no cytokine and growth factors at 20% of normal levels. After 24 h of treatment, cells were dissociated and stained according to the markers in Table 1 and assessed using flow cytometry. Averages are shown as a percent of the total population. Comparisons are noted with the *p*-value between the groups; *n* = at least three cultures per groups. ^*^p < 0.05 by Student's *t* test and Tukey's post hoc test.

BNAP, bipotential neuron astrocyte progenitor; EdU, 5-ethynyl-2′-deoxyuridine; FGF, fibroblast growth factor; GRP2, glial restricted progenitor 2; GRP3, glial restricted progenitor 3; MP1, multi-potential progenitor-1; MP2, multi-potential progenitor-2; MP4, multi-potential progenitor-4; NSC, neural stem cell; P, post-natal day; PFMP, platelet derived growth factor and FGF-2 responsive multi-potential progenitor; ProK2, prokineticin-2.

**Table 5.** Average Percentages of Proliferating Neural Cells and Their Corresponding P-values after 24 h of ProK2 Treatment or No Cytokine Treatment

	EdU+					
Group	O4+	O4–/CD133+	Glast+	Glast+/CD133–	Glast+/CD15+	Glast+/CD15–
No cytokine average	10.897	3.191	27.101	19.216	1.513	16.121
ProK2 average	8.277^*^	1.884	14.078^*^	12.987^**^	0.509^*^	14.327

Neural tissue rich in SVZ cells were collected from P4 pups and neurosphere cultures were established. Spheres were propagated for several days, passaged, and grown for an additional 5 days. Spheres were starved of growth factors for 15 h and growth factors were reintroduced along with EdU and 1 × 10^−8^ M ProK2 or no cytokine and growth factors at 20% of normal levels. After 24 h of treatment, cells were dissociated and stained according to the markers in Table 1 and assessed using flow cytometry. Averages are shown as a percent of the total population. Comparisons are noted with the *p*-value between the groups; *n* = at least three cultures per group. ^*^p < 0.05, ^**^p < 0.01 by Student's *t* test and Tukey's post hoc test.

EdU, 5-ethynyl-2′-deoxyuridine; P, post-natal day; ProK2, prokineticin-2; SVZ, subventricular zone.

### ProK2 is produced by neurons in the injured neocortex

**FIG. 5.**
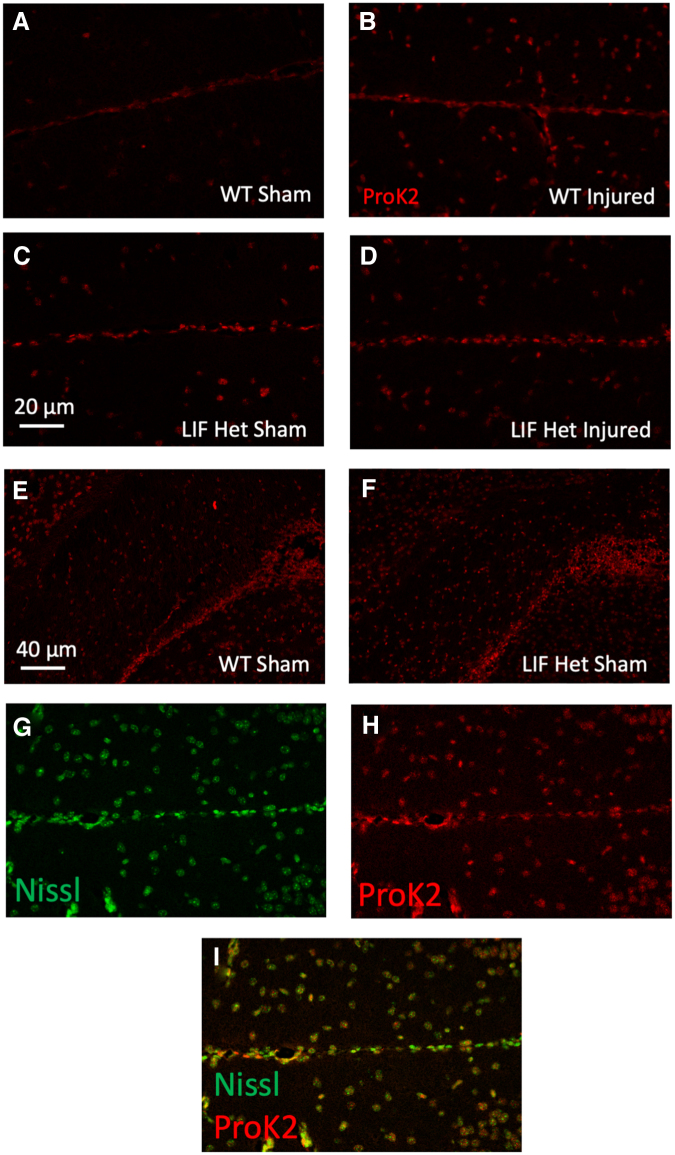
ProK2 is most abundant in WT injured mice and in neurons. P20 mice were injured by CHI and perfused 48 h later. Brains were sectioned at 6 μm and stained for ProK2 (red). Representative images are shown of ProK2 of the midline, injured neocortex, below the center of the impact zone 2DPI from **(A)** WT sham, **(B)** WT injured, **(C)** LIF Het sham, and **(D)** LIF Het injured mice. Representative images are shown of ProK2 of the SVZ at 2DPI from **(E)** WT sham and **(F)** LIF Het sham mice. Representative channel specific images are shown of the injured midline, neocortex of WT injured mice for **(G)** Nissl (green), **(H)** ProK2 (red), and **(I)** merged. Data are representative of five individual mice. CHI, closed head injury; DPI, days post-injury; Het, heterozygous; LIF, leukemia inhibitory factor; P, post-natal day; ProK2, prokineticin-2; SVZ, subventricular zone; WT, wild-type.

To date, it has not been established which cells produce ProK2 in the neocortex; therefore, we used *in situ* hybridization (RNAScope) to visualize the expression of ProK2 in the control and CHI mouse brain. We used a red fluorescent ProK2 probe and a green, fluorescent Nissl counterstain. At 48 h of recovery from the CHI, there was increased ProK2 mRNA fluorescence in WT injured mice (Fig. 5B) when compared with LIF Het injured mice (Fig. 5D) and sham controls (Fig. 5A,C). There was no qualitative difference in ProK2 expression between the WT and LIF Het mice, but there was a trend for ProK2 fluorescence to be lower in the injured LIF Het mice, which is consistent with qPCR data from Figure 3B.

Although ProK2 is a small molecule that can diffuse from the injured neocortex to affect the cells in the SVZ, it was of interest to establish whether ProK2 is produced within the SVZ itself. From the same sections processed for ProK2+ RNAScope, we observed that ProK2 was highly expressed within the SVZ; however, there were no obvious differences between WT sham (Fig. 5E) and LIF Het sham (Fig. 5F) mice. As the brains had been stained for ProK2 (red; Fig. 5G) and Nissl (green; Fig. 5H), we observed that the majority of ProK2 staining overlapped with large diameter Nissl-stained cells (Fig. 5I), consistent with the interpretation that neocortical neurons are the primary source of ProK2 in both the uninjured and the injured brain.

### LIF addition to neurosphere cultures increases levels of ProK2

**FIG. 6.**
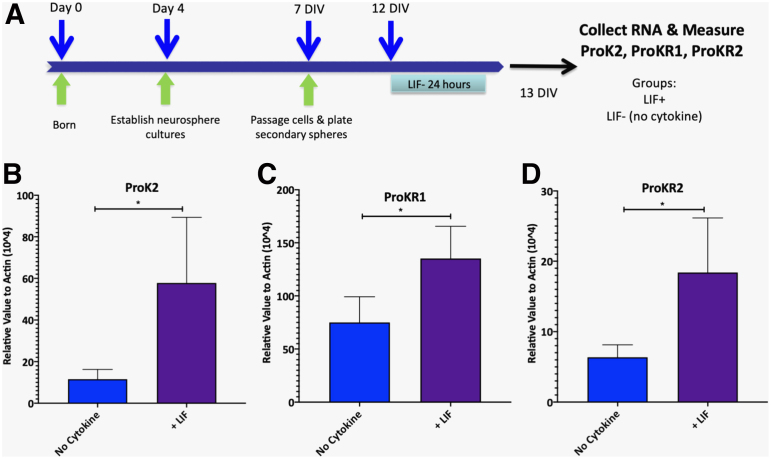
LIF directly affect levels of ProK2 and its receptors in neural progenitors. **(A)** Secondary neurospheres were established and LIF was applied at 5ng/mL for 24 h. mRNA was isolated; cDNA was synthesized and levels of ProK2 and its receptors were measured by qPCR. **(B)** ProK2 mRNA, **(C)** ProKR1 mRNA, and **(D)** ProKR2 mRNA. Data represent means ± SD from four independent cultures per group. Statistical significance was evaluated by Student's *t* test. *p < 0.05. cDNA, complementary DNA; LIF, leukemia inhibitory factor; mRNA, messenger RNA; ProK2, prokineticin-2; qPCR, quantitative polymerase chain reaction; SD, standard deviation.

As mRNA levels of ProK2 dramatically increased in WT injured mice and as this increase appeared to require LIF (Fig. 3B, Table 3), we hypothesized that LIF was directly increasing the levels of ProK2. Therefore, we evaluated the levels of the ProK2 ligand as well as its receptors, ProKR1 and ProKR2, in neurospheres treated with LIF for 24 h by qPCR (Fig. 6A). As predicted, LIF increased the mRNAs for ProK2 (Fig. 6B), ProKR1 (Fig. 6C), and ProKR2 (Fig. 6D). These data are consistent with the interpretation that LIF directly affects ProK2 levels and its receptors' levels in the neural progenitors of the SVZ.

## Discussion

In the experiments described in this article, we used several complementary approaches to determine how a mild CHI affected the proliferation of the astrocyte progenitors within the SVZ as well as whether their proliferation required LIF. We produced a mild CHI to the neocortex of P20 mice and performed immunofluorescence and flow cytometric analyses to identify and quantify the proliferation of distinct SVZ neural progenitors. Our studies demonstrate that: 1) astrocyte progenitor cell proliferation positively correlates with LIF expression, but NSC proliferation does not; 2) changes in astrocyte proliferation can be attributed to LIF and not to levels of other cytokines or growth factors; 3) ProK2 expression increases 5-fold in response to a CHI in WT mice; 4) LIF increases ProK2 expression and the injury-induced increase in ProK2 requires LIF; 5) ProK2 is highly expressed in the SVZ, whereas cortical neurons are primarily responsible for producing ProK2 in the neocortex; and 6) ProK2 suppresses the proliferation of primitive SVZ progenitors.

Studies have shown that SVZ cells can migrate to other regions of the brain beyond the olfactory bulb after an injury and most migration studies have followed the paths taken by the doublecortin+ neuroblasts with less consideration given to the paths taken by the glial progenitors.^32–40^ But in the immature brain, there is extensive migration of SVZ glial progenitors.^41^ Moreover, with elevated levels of cytokines and chemokines produced in the injured brain, there are increased numbers of progenitors exiting the SVZ that contribute to remodeling and repair processes. Indeed, Goings and colleagues^42^ showed that after an aspiration lesion of the neocortex there was robust emigration of retrovirally labeled SVZ cells toward the injury. Those cells that migrated from the SVZ to the CC differentiate into oligodendrocytes, whereas those that migrated toward the lesioned neocortex differentiated into astrocytes.^42^

In light of those data and our previous report that showed that abated astrogliosis in LIF Het mice in response to pediatric CHI,^6^ we postulated that the decreased astrogliosis might be due, in part, to an inhibited proliferative response of the SVZ astrocyte progenitors. Supporting that hypothesis, we report that LIF Het injured mice demonstrate reduced levels of proliferating astrocyte progenitors in the SVZ following a CHI. However, we also found that astrocyte lineage cells proliferate less in the WM of the LIF Het mice after CHI, indicating that both the parenchymal astrocyte lineage cells and the astrocyte progenitors within the SVZ require LIF to begin to divide after injury.

In the studies described here and in a companion article,^43^ we found that the bona fide NSCs are not proliferating in response to this mild injury, as they do in more severe injury models.^12^ We used Glast to identify the astrocyte progenitors by flow cytometry. Glast is a glutamate transporter and although it primarily marks astrocytes, it also can label NSCs. Therefore, to differentiate NSCs from glial progenitors, we used an 8-color flow panel that has the capability to identify and quantify the relative abundance of 14 distinct SVZ neural precursors.^29^ Using this same flow panel, Goodus and associates^12^ reported a 2-fold increase in NSCs in the SVZ of the ipsilateral hemisphere of CCI injured mice at 24 h of recovery. However, when we used this same flow panel to evaluate the NSCs after CHI, we did not observe any changes in the proportions of NSCs in the SVZ or in the percentage of EdU+ cells. These data support the conclusion that a single CHI is not sufficient to activate the NSCs of the SVZ,^43^ likely because it is much less severe.^43^ However, a limitation to these studies is that we have only evaluated the NSCs at 24, 48, and 96 h of recovery from the CHI. As the NSCs have a very slow cell-cycle time, it is possible that they are activated and start to proliferate but they are doing so outside the time-points that we have evaluated.

Astrocytes are key regulators of homeostasis and neuronal health and become activated after CNS injuries. Cytokine-activated astrocytes produce energy substrates and trophic factors, scavenge free radicals and excess glutamate, actively restore the blood–brain barrier, promote neovascularization, restore ionic homeostasis, and promote remyelination.^44,45^ Activated astrocytes extend their processes to neighboring injured neurons to promote their survival and to preserve synapses, and their ability to do so requires the activation of signal transducer and activator of transcription-3 (STAT-3).^46^ Indeed, selectively deleting STAT-3 from astrocytes prevented the astrocytes from surrounding inflammatory cells that in turn, increased the area of neuronal loss after a spinal cord injury.^47^ Deleting STAT-3 in astrocytes also produced more widespread inflammation after TBI.^48–51^ Because STAT-3 is activated by the IL-6 family of cytokines that includes LIF, and because LIF increases ∼15-fold after a CNS injury, the decrease in astrocyte activation and the increase in cellular damage after a CHI in the LIF Het mice are likely due to decreased STAT-3 activation in the astrocytes; however, this remains to be demonstrated.

Our analysis of ProK2 was informed by previous studies that showed that ProK2 increased proliferation of cultured rat astrocytes.^31^ However, in our studies, ProK2 had a profound anti-proliferative effect. Although it is possible that there are species-specific differences in responses to ProK2, it is more likely that differences in the state of maturation of these progenitors can explain the different responses. Notably, Neal and colleagues^31^ used a GFAP-green fluorescent protein (GFP) reporter and found that GFAP+ cells *in vivo* increased in number with ProK2 treatment. However, we found that applying ProK2 *in vitro* decreased the number of proliferating astrocyte progenitors.^31^ Therefore, we conclude that ProK2 exerts an anti-proliferative effect on the primitive neural progenitors of the SVZ, whereas it acts as a mitogen for immature and potentially mature astrocytes.

ProK2 exerts both proliferative as well as chemotactic effects on SVZ cells. During development, ProK2 is a chemokine that directs migrating neuroblasts to the olfactory bulb.^52^ This action is likely through the ProKR2, as this receptor is found in the SVZ, olfactory bulb, rostral migratory stream, and dentate gyrus.^52^ Consistent with ProK2 having a regulatory function on SVZ cells, we found that ProK2 mRNA was abundantly expressed in the SVZ of both injured and uninjured mice.

Langley and co-workers^53^ found that ProK2, and a ProK2 agonist, increased SVZ cell proliferation in brain slices. Similarly, lentiviral overexpression of ProK2 increased proliferation and differentiation in these brain slice cultures,^53^ leading these authors to conclude that ProK2 enhanced NSC proliferation and differentiation. However, when we applied ProK2 to neurospheres, we found that the number of NSCs was not significantly affected. But one key difference between these two studies is how the NSCs were defined. Langley and co-workers defined any BrdU+ cell in the SVZ as an NSC, but the SVZ is a heterogenous mix of progenitors and stem cells, all of which can incorporate BrdU. On the other hand, we used a multi-color flow panel that used eight markers to identify true NSCs as well as 13 other progenitors. ProK2 did not increase the proliferation of any neural progenitor subtype within the neurospheres. Based on these data and those from others, we conclude that ProK2 is not stimulating the proliferation of the NSCs, but that it is stimulating the proliferation of immature astrocytes.

Brain injuries trigger a cascade of pathophysiological processes that ultimately lead to cell death, and our data reveal a potentially important role for ProK2 during recovery from a TBI. In addition to its role in neural development, ProK2 has well-documented actions as neurotrophic factor for dopaminergic neurons, where it protects against oxidative stress, mitochondrial dysfunction, and degeneration.^54^ Other studies using models of cerebellar excitotoxicity and ischemia show that ProK2 is neuroprotective.^55,56^ Fate mapping studies have shown that cells migrate from the SVZ to the injured cortex after TBI. Other studies have shown that ProK2 is induced after injury,^57,58^ where it has been suggested that ProK2 can promote the migration of new neurons toward the injury site following TBI.^59^

Supporting the role for ProK2 in this process, injecting ProK2 into the neocortex of uninjured mice attracts SVZ cells and administering a ProKR2 antagonist decreases the number of SVZ cells that reach the injured cortex.^22^ Although these studies demonstrate that ProK2 participates in the emigration of neural progenitors from the SVZ, our results are the first to demonstrate that the increase in ProK2 expression requires LIF. Both LIF and ProK2 receptors are druggable and can be stimulated to enhance cell survival after TBI. Accordingly, our future studies will evaluate the neuroprotective and neuroregenerative effects of administering LIF of ProK2 alone or together to promote better recovery of the acutely injured brain.

## Conclusion

In the experiments described here, we show that a single CHI affects the proliferation of astrocyte progenitors within the SVZ and that this process requires LIF. These data reinforce the view that LIF is an essential injury-induced cytokine following a pediatric TBI, whose function is to coordinate the glial neuroprotective response to brain injury. These data also reveal a new role for LIF as an essential regulator of the neurotrophic factor PRoK2.

## Abbreviations Used

ANOVA: analysis of variance
CC: corpus callosum
CCI: controlled cortical impact
cDNA: complementary DNA
CHI: closed head injury
CNS: central nervous system
DMEN: Dulbecco's modified Eagle's medium
DPI: days post-injury
EdU: 5-ethynyl-2′-deoxyuridine
EGF: epidermal growth factor
ELISA: enzyme-linked immunosorbent assay
FGF: fibroblast growth factor
GFAP: glial fibrillary acidic protein
GRP: glial restricted progenitor
HEPES: Times New Roman
Het: heterozygous
IL: interleukin
LIF: leukemia inhibitory factor
MP2: multi-potential progenitor-2
mRNA: messenger RNA
NIH: National Institutes of Health
NSC: neural stem cell
OCT: optimal cutting temperature
P: post-natal day (e.g., P20)
PBS: phosphate-buffered saline
PFMP: platelet derived growth factor and FGF-2 responsive multi-potential progenitor
PI: post-injury
ProK1: prokineticin-1
ProK2: prokineticin-2
ProKR1: prokineticin-1 receptor
ProKR2: prokineticin-2 receptor
qPCR: quantitative polymerase chain reaction
RIPA: radioimmunoprecipitation
SD: standard deviation
STAT-3: signal transducer and activator of transcription-3
SVZ: subventricular zone
TBI: traumatic brain injury
WM: white matter
WT: wild-type
